# Increased Vulnerability to Pregnancy and Sexual Violence in Adolescents with Precocious Menstruation

**DOI:** 10.1155/2020/5237814

**Published:** 2020-03-11

**Authors:** Giani Silvana Schwengber Cezimbra, Dioclécio Campos Junior, Edward Araujo Júnior, Cristina Aparecida Falbo Guazzelli, Álvaro Nagib Atallah

**Affiliations:** ^1^Department of Pediatrics, Faculty of Medical Sciences-University of Brasília (UnB), Brasília-DF 70.910-900, Brazil; ^2^Department of Obstetrics, Paulista School of Medicine-Federal University of São Paulo (EPM-UNIFESP), São Paulo-SP 04021-001, Brazil; ^3^Discipline of Evidence-Based Medicine, Paulista School of Medicine-Federal University of São Paulo (EPM-UNIFESP), São Paulo-SP 04024-002, Brazil

## Abstract

This cross-sectional, observational, and descriptive study was conducted to evaluate the association between age at menarche in the adolescent population and the age at sexual initiation, age at first pregnancy, and experience of sexual violence in the adolescent population visiting a primary health unit in Brazil. We recruited 201 female adolescents who visited the gynecology outpatient clinic of a Basic Health Unit in the Federal District of Brazil. These adolescents answered a questionnaire with regard to sexual and reproductive health during doctor's appointments. To calculate the association, we recorded data for age at menarche, age at first sexual intercourse, age at first pregnancy, and experience of sexual violence. Pearson and Mann–Whitney correlation coefficient statistical tests were performed to evaluate the association between these variables. Mean age at menarche was lower among adolescents who became pregnant (*p* = 0.0004) and those who experienced sexual violence (*p* = 0.0008). Further, there was a strong association between age at menarche and age at first sexual intercourse (*p* < 0.0001). This study also demonstrated that the earlier the age at menarche, the earlier was the age at sexual initiation and age at first unintended pregnancy and the greater was the risk of experiencing sexual violence. Early menarche may be considered a vulnerability factor during adolescence.

## 1. Introduction

Menarche, or the first menstrual bleeding, is a significant event in the reproductive life of a woman. The onset of menarche is preceded by a complex cascade of hormonal changes during puberty [1]. Hormonal regulation of sexual maturation is susceptible to several factors from the beginning of prenatal life. Early pubertal development induces medical and social problems and, eventually, results in increased morbidity and mortality in adulthood. Age at menarche is one of the most significant features commonly used for retrospective epidemiological studies on female sexual maturation in a population [1].

According to Karapanou and Papadimitriou [2], variability in the date of the first menstruation is due to complex interactions between genetic characteristics and environmental factors. Genetic factors contribute to age at menarche by approximately 57–82% [2, 3]. These factors include prenatal and postnatal determinants of menarcheal age, such as ethnic differences, maternal history, maternal weight gain during pregnancy, gestational diabetes, prematurity, and birth weight [4, 5].

Environmental factors have attracted increasing attention because many of them are potentially controllable and are related to climate, geographic location, nutritional status, socioeconomic level, education, ethnicity, number of children in the family, and other variables [2].

Other factors such as family disruption, childhood adversity, and ongoing stress may also accelerate a girl's sexual and reproductive development. Adverse childhood experience such as sexual abuse and presence of psychological disorders such as autistic spectrum, Down syndrome, and cerebral palsy may cause variation in the onset of menses [5–9].

Yermachenco and Dvornyk [1] state that general improvement in the socioeconomic and health conditions of populations has resulted in an earlier onset of puberty and the anticipated onset of menses. These conditions impact the nutritional and psychosocial state of girls during childhood and adolescence. In this way, early menarche may be considered a positive trend with regard to improvements in socioeconomic conditions and wellbeing in countries where hunger, drought, and local conflicts continue to be serious problems [10].

Studies conducted in developed countries suggest that early menarche (at the age of 11 years or below) increases the vulnerability of these adolescents to negative sexual and reproductive health outcomes including early sexual initiation, early pregnancy and delivery, and sexual violence [1, 11–13].

This study is aimed at evaluating the association between age at menarche and age at sexual initiation, age at pregnancy, and experience of sexual violence in the adolescent population visiting a primary health unit in the Federal District, Brazil. Establishing this relationship may help contribute to preventive strategies, protective health interventions, and the sexual and reproductive rights of adolescents.

## 2. Materials and Methods

This clinical cross-sectional, observational, descriptive study was conducted among low-income female adolescents who sought care by spontaneous demand and visited the gynecology outpatient clinic in a Basic Health Unit (BHU), Sobradinho, Federal District, Brazil. The sample consisted of 201 female adolescents, aged 10–19 years, who attended the BHU for adolescents for 12 months. The project was approved by the Research Ethics Committee of the Faculty of Medicine at the University of Brasília and registered under number 119/2007. In addition, a duly completed and signed consent form was received from the participants and their guardians for those under 18 years of age, to accept participation in the study and the delivery of the Terms of Free and Informed Consent.

Inclusion criteria were age between 10 and 19 years, female gender, previous menarche, and those who agreed to participate in the study. Exclusion criteria were presence of any disease or use of medications that could interfere with sexual maturation.

The questionnaire was composed of direct questions with yes or no answers or related numbers, such as in the case of the participants' age. A database was then constructed with the information of the following variables: age at menarche (independent variable), age at sexual initiation, age at first pregnancy, and experience of sexual violence in the adolescents' dependent variables. The answers in the questionnaire were exclusively filled by the researcher for questions directed to the adolescents, who answered freely and independently. Questions on sexual violence required direct, affirmative (yes) or negative (no) answers after clarifying the concept. In Brazil, sexual activity with girls under the age of 14 years is legally considered sexual violence [14, 15].

The database was constructed using Excel 2000 software (Microsoft Corp., Redmond, WA, USA). Statistical tests such as ANOVA and Mann–Whitney tests were performed using SAS software version 8.02 (SAS Institute Inc., Cary, NC, USA), and a mathematical model of linear regression was established.

Pearson correlation coefficients (*r*) were also used to quantify the degree of association between two variables. Initially, each variable was analyzed individually, and then statistical analysis was performed to access the association between the independent variable and the other dependent variables by crossing the data of age at menarche with the data of age at first sexual intercourse, age at first pregnancy, and occurrence of sexual violence. To verify this, frequencies, averages, standard deviations, and confidence intervals (CIs) were calculated. Statistical significance at *p* < 0.05 was used for all analyses.

## 3. Results

At the BHU, 768 adolescents sought services during the 12 months of evaluation, of them, 201 agreed to participate and answered the questionnaire. The mean age of the adolescents during care was 16.9 years. Of the 201 adolescents, 191 (95%) were aged 15 years or older at the time of the consultation. The mean age at menarche was 12.1 years, and of the 201 participants, 135 (67%) menstruated before 12 years of age and 66 (33%) after 13 years of age.

Of the 201 adolescents, 171 (85%) reported having initiated sexual activity at the time of visiting the BHU. The mean age at first sexual intercourse was 15 years. Of those 171 participants, 58 (34%) reported having started their sexual life at 14 years or younger and 21 (12%) began at the age of 15 years. For 92 (54%) of the adolescents interviewed, sexual initiation occurred at the age of 16 years.

**Figure 1:**
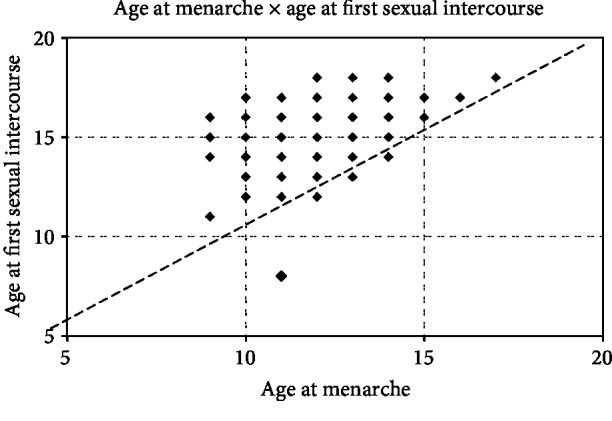
Correlation between the age at menarche and age at first sexual intercourse of adolescents attended at a Basic Health Unit (age at menarche × age at first intercourse).

During the evaluation of the findings, we observed that age at first sexual intercourse is usually after menarche and that the later the age at menarche, the later is the age at first sexual intercourse (Figure 1). These data show an association between age at menarche and age at first sexual intercourse. The Pearson correlation coefficient (*r*) was 0.38, and it indicated a moderate positive association between the two sets of information (*p* < 0.0001).

Among the 201 adolescents, 114 (56.7%) had already become pregnant, and the mean age was 16.2 ± 1.5 years for the first pregnancy. The CI was 15.9–16.5 years for the first pregnancy, ranging from 11 to 18 years (Table 1).

**Figure 2:**
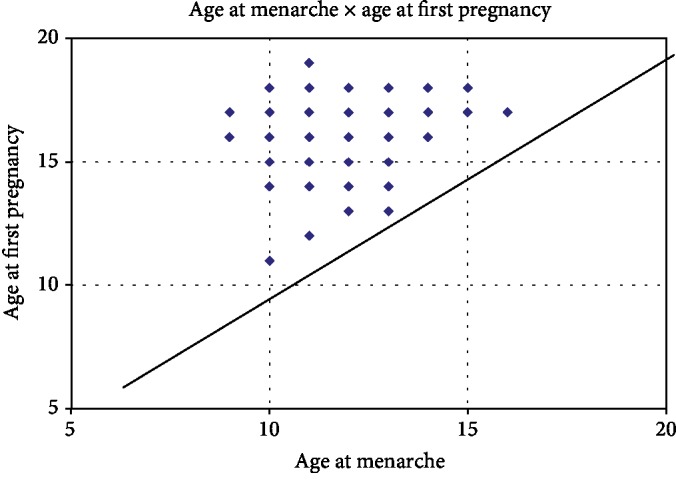
Correlation between the age at menarche and the age at first pregnancy of adolescents attended at a Basic Health Unit (age at menarche × age at first pregnancy).

Among the adolescents, 30 (26.3%) became pregnant at the age of 15 years or younger. Age at menarche and age at first pregnancy showed a significant association (*p* = 0.0004). Thus, age at menarche also exerted influence on age at first pregnancy in this group (Figure 2).

The number of respondents who answered the questions on sexual violence was 103. Of these 103 participants, 51 (49%) experienced some form of sexual violence. There were 28 declared cases of sexual violence, and in 23 cases, there was a presumption of violence, as these adolescents were under 14 years of age. Experience of sexual violence was also significantly associated with early menarche, with a mean age at menarche of 11.5 years. This age was different from that of participants with negative answers for this event, in which the mean age at menarche was 12.2 years. The age difference in the onset of menarche among adolescents who experienced sexual violence was statistically significant (*p* < 0.05) (Table 2).

The statistical model, as shown in Table 3, provided details on which factors were related to age at menarche. This model provided details on the association of age at first sexual intercourse and age at first pregnancy with age at menarche (*R*^2^ = 0.9865).

## 4. Discussion

The results of this study corroborate the literature, confirming that there is a progressive tendency toward precocity of sexual maturation. This is an important medical and social problem, as it can result in increased morbidity and mortality in adult life [1–3]. According to the data obtained in the study, age at menarche predominated at approximately 12 years. It is estimated that during the twentieth century, age at menarche fell by approximately 3 months per decade [2, 16, 17]. This universal phenomenon can result in higher risks such as early sexual initiation, unplanned pregnancy, and sexual violence in girls and adolescents [1, 11–13]. Currently, age at menarche is considered a reliable marker among the other positive changes that occur in pubertal development [2, 18, 19].

The data obtained from the group showed that the earlier the menarche, the earlier the sexual life begins. Among all associations, the relationship between early menarche and the earliest onset of sexual activity has the greatest impact (*p* < 0.0001). The data in the literature are similar to ours', and because of psychoemotional immaturity, misinformation, and lack of access to contraceptives, there is a high risk of pregnancy and sexually transmitted diseases [13].

The precocity of age at menarche may be associated with other consequences such as health problems at a later age, presence of risk behaviors, early sexual initiation, smoking, breast cancer, diabetes, impaired fertility, cardiovascular diseases, obesity, and psychological disorders. Data from other studies corroborate the data of this study by identifying that early menarche (at the age of 11 years or less) increases the vulnerability of adolescents to negative sexual health outcomes and reproductive health in high-income countries. This includes early sexual initiation, early pregnancy and childbirth, and sexual violence [10–13].

A study conducted in New Zealand demonstrated findings similar to ours in girls who reached menarche at 10–11 years of age were significantly more likely to be pregnant at 18 years of age than those who reached menarche at a later age [11]. In studies conducted among young women in the United States, age at menarche was associated with the initiation of sexual life, which, in turn, correlated directly and positively with age at first pregnancy [10, 13].

In the population evaluated in this study, 114 (56.7%) adolescents had already become pregnant at least once, and 10% of them, two or more times. Mean age at first pregnancy was 16.2 years and our results showed an association between first pregnancy and early menarche. A major concern among teenagers is the risk of recurrence of pregnancy. A review of the American literature showed that 35% of low- and middle-income adolescents became pregnant again in less than 2 years [20]. The same trend was seen in another study [21].

An experience of sexual violence was confirmed by 61 (49%) adolescents in the evaluated group (considering the presumed violence). This study showed a significant association between experiencing sexual violence and early menarche, corroborating the findings in the literature [13, 15]. According to a study conducted by Wise et al., sexual violence was positively associated with early menarche, with an increase in the frequency of sexual abuse cases [15]. In this study, a weak but statistically significant association between physical violence and early menarche was observed. The association between sexual violence and early menarche was stronger when menarche occurred before the age of 11 years.

Although the data are consistent with the data reported in the literature, longitudinal studies with a larger number of participants are required to elucidate the association of these findings.

Early sexual maturation is not always accompanied by psychoemotional and social maturity, which hinders autonomy for self-care and protection as well as restricts access to resources for living in a healthy and safe manner. It can definitively compromise life projects in adolescence and adulthood. It is possible, therefore, to associate early menarche with a greater frequency of unprepared and unprotected sexual initiation in relation to various biopsychosocial aspects, unintended pregnancy in adolescence and experiencing sexual violence, and whether the violence is declared by the adolescent or not yet perceived by them but presumed by justice and pseudopermitted by society.

Investing in research on sexual and reproductive health is an effective strategy to identify the factors associated with the increased risks that adolescents face concerning sexual violence, unintended pregnancies, and other diseases and injuries to which they are vulnerable. The data obtained will allow the development of necessary and effective measures to protect and positively influence a new generation of children and adolescents. Thus, this research will assist them in achieving their goals, fulfilling their aspirations, guaranteeing their rights, and achieving an overall improved quality of life.

## Figures and Tables

**Table 1:** Distribution of the age at the first pregnancy of adolescents attended at a Basic Health Unit.

Age at first pregnancy	*N*	%
11	1	0.88
12	2	1.75
13	2	1.75
14	10	8.77
15	15	13.16
16	34	29.82
17	26	22.81
18	24	21.05
Total of respondents	114	100

^∗^Nonrespondents: 87 (30 without sexual activity so far and 57 who never got pregnant).

**Table 2:** Correlation between age at menarche and the experience of sexual violence among adolescents attended at a Basic Health Unit.

Sexual violence	*N*	Mean	Standard deviation	Confidence interval	*p* ^∗^
Yes	51	11.5	1.2	(11.1–11.8)	0,0008
No	62	12.2	1.3	(11.9–12.6)	

^∗^Mann–Whitney test.

**Table 3:** Linear regression model between age at menarche and age at first pregnancy among adolescents attended at a Basic Health Unit.

Age at menarche = 0.3935 (age at first sexual intercourse) + 0.3825 (age at first pregnancy) − 0.0034			
Variable		Parameter	*p*
Age at first intercourse		0.39346	0.0049
Age at first pregnancy		0.38254	0.003

^∗^Linear regression model without intercept.

## Data Availability

The data used to support the findings of this study are available from the corresponding author upon request.
